# Translation, Validity, and Reliability of Mental Health Literacy and Help-Seeking Behavior Questionnaires in Indonesia

**DOI:** 10.3389/fpsyt.2021.764666

**Published:** 2022-01-18

**Authors:** Fransiska Kaligis, R. Irawati Ismail, Tjhin Wiguna, Sabarinah Prasetyo, Wresti Indriatmi, Hartono Gunardi, Veranita Pandia, Kusuma Minayati, Clarissa Cita Magdalena, Garda Widhi Nurraga, Muhammad Fariz Anggia, Subhan Rio Pamungkas, Thach D. Tran, Marjo Kurki, Sonja Gilbert, Andre Sourander

**Affiliations:** ^1^Doctoral Program in Medical Sciences, Faculty of Medicine, Universitas Indonesia, Jakarta, Indonesia; ^2^Department of Psychiatry, Faculty of Medicine Universitas Indonesia, Cipto Mangunkusumo Hospital, Jakarta, Indonesia; ^3^Department of Child Psychiatry, University of Turku, Turku, Finland; ^4^Faculty of Public Health, Universitas Indonesia, Depok, Indonesia; ^5^Department of Dermatovenereology, Faculty of Medicine Universitas Indonesia, Cipto Mangunkusumo Hospital, Jakarta, Indonesia; ^6^Department of Child Health, Faculty of Medicine Universitas Indonesia, Cipto Mangunkusumo Hospital, Jakarta, Indonesia; ^7^Department of Psychiatry, Faculty of Medicine Universitas Padjajaran, Hasan Sadikin Hospital, Bandung, Indonesia; ^8^Department of Psychiatry, Faculty of Medicine Universitas Syiah Kuala, Aceh, Indonesia; ^9^Public Health and Preventive Medicine, Monash University, Melbourne, VIC, Australia

**Keywords:** mental health literacy, help-seeking behavior, adolescents, validity, reliability

## Abstract

**Background and Aim:** Mental health is an integral part of adolescent wellbeing. However, only few adolescents understand the importance of mental health and are aware of the right time to seek help. Lack of knowledge and stigma may impede help-seeking behavior. To assess these aspects, three questionnaires have been developed in the English language. This study aims to assess the validity and reliability of an Indonesian version of the Mental Health Literacy and Help-Seeking Behavior set of questionnaires among adolescents in Indonesia.

**Methods:** This is a cross-sectional study that used The Mental Health Literacy and Help-Seeking Behavior set of questionnaires developed by Kutcher and Wei. The set consists of three questionnaires: the Mental Health Knowledge, Attitude Toward Mental Health, and Help-Seeking Behavior questionnaire. The study was conducted between October 2020 and January 2021 with 68 first-year medical students at the University of Indonesia, who represented adolescents in a transitional phase. The questionnaires were translated into the Indonesian language by a bilingual psychiatrist and reviewed by 10 expert psychiatrists to determine content validity [Item-Level Content Validity Index (I-CVI) and Scale-Level Content Validity Index (S-CVI)]. Cronbach's alpha values were used to assess internal consistency (reliability).

**Results:** The content validity test produced positive results with an I-CVI scores of 0.7–1.0 and S-CVI scores of 0.87, 0.90, and 0.99 for the knowledge, attitude, and help-seeking behavior questionnaires, respectively. For the reliability test, Cronbach's alpha values were 0.780 for the attitude questionnaire and 0.852 for the help-seeking behavior questionnaire, while the value for the knowledge questionnaire was 0.521.

**Conclusion:** The ability to properly measure mental health through the availability of accessible, valid, and understandable tools plays an important role in addressing mental health issues among adolescents. In the current study, the Indonesian translations of all three questionnaires examining knowledge, attitude, and help-seeking behavior were considered to be valid and reliable.

## Introduction

Adolescents suffer from mental health problems just like everyone else. Approximately 18% of the Indonesian population suffers from several types of mental disorders (1). Wiguna et al. (2) demonstrated that 33.5% of adolescents who sought help exhibited mood disorders. Self-harm is also prevalent among adolescents, reaching its peak around puberty. Nevertheless, adolescents do not always seek help. One study indicated that only one in four adolescents with depression or one in three adolescents with a mental illness sought help, whereby those who did otherwise may be at risk of drug abuse, risky behavior, decreased quality of life, and lowered life expectancy (3–6). Adolescents may develop mental health problems when facing the experience of transitioning to young adulthood. Their mental health may be affected not only by their biological or executive function development but also by their social environments, for example, family, peers, and schools (7). Adolescents transitioning to adulthood may also need to adapt to new situations and challenges as they pursue higher education. Entering college may promote a sense of accomplishment. However, there may also be new stressors that could trigger mental distress (8).

The recent World Mental Health Surveys conducted by the World Health Organization revealed that 20.3% of college students had 12-month mental health disorders assessed with Diagnostic and Statistical Manual of Mental Disorders, fourth edition (DSM-IV)/Composite International Diagnostic Interview. Of that group, 83.1% of the students experienced mental health disorders before enrolling in college. Those who experienced the onsets of mental health disorders before entering college had a higher chance of dropping out. In addition, the study revealed that only 16.4% of the students with 12-month disorders had received mental healthcare treatment (9). Among college students, medical students have been reported to have higher rates of perceived stress and emotional distress (10). Atkinson (11) found that one-third of medical students showed stress and depressive symptoms, while half of the students were anxious upon entering their first year. Adolescents entering medical schools while transitioning to adulthood may need to be assessed for their mental health knowledge, attitudes to mental health, and their help-seeking behavior to develop specific measures of early intervention to address their psychological distress and promote help-seeking behavior.

Being able to recognize specific mental illnesses, knowing how to obtain information about treatment and adequate professional help, and performing help-seeking behavior are crucial. One study revealed that among its 2,000 subjects, only 39% were able to identify symptoms of depression, and only 27% were able to identify symptoms of schizophrenia (12, 13). Another study involving adolescents found that only 16.4% of respondents could be classified as adequately informed to identify and intend to seek help for certain issues (14). In 2007, the World Health Organization (WHO) defined health literacy as the cognitive and social skills that determine the motivation and ability of individuals to gain access to, understand, and use information in ways that promote and maintain good health. Mental health literacy was defined as the understanding of how to obtain and maintain good mental health, understanding mental disorders and their treatments, developing capacities to decrease stigma, and developing capacities to enhance help-seeking efficacy (15).

The most powerful determinant of actual help-seeking behavior is help-seeking intentions, with studies demonstrating that help-seeking attitudes could predict help-seeking intentions. Sub-factors of help-seeking attitudes are recognition of the need for help, stigma tolerance, interpersonal openness, and therapist confidence (3). The attitude shown by adolescents toward mental health is important, as overall negative attitudes toward mental health may impede help-seeking behavior. Penn et al. (16) demonstrated that adolescents and young adults had greater mental health knowledge, yet they believed those with mental illnesses to be different to those who are “normal.”

There are several factors that facilitate or impede help-seeking behavior: a preference to look to informal sources, e.g., friends and family; a fear of others reacting to their story; and a lack of knowledge of where to seek help (17). Another study found impeding factors related to help-seeking behavior to include a lack of information; biased recognition of severity, cost, shame, time, and distance; and distrust in healthcare professionals (18). According to another study, factors determining help-seeking behavior in adolescents included perceived level of benefit, general health motivation, extraversion, social support, and perceived barriers (cost) (19). Mental health knowledge and attitudes and help-seeking behavior must be assessed to understand the capacity of individuals to seek help, including its accessibility.

Therefore, a comprehensive understanding of mental health literacy is essential for more effective and targeted interventions or programs to address the rise in mental health issues among adolescents (20, 21). Subsequently, utilizing proper and relevant tools would be beneficial in obtaining correct measurements that portray the latest information regarding mental health literacy in adolescence (20). Nevertheless, recent studies on mental health literacy have used limited measures focusing on specific subpopulations. Sorensen et al. (22) mentioned a widely used instrument called the Health Literacy Survey European Questionnaire 47 (HLS-EU-QS47), which assesses a broad spectrum of mental health literacy topics. In Indonesia, there was a self-reporting questionnaire called Willingness to Seek Professional Counseling Outside the university (WSPCO), which was used as a tool to obtain data regarding contributing factors impeding help-seeking behavior to reach out for psychological help and facilities (18). However, both questionnaires feature more general items and do not portray the division of mental health literacy into three aspects: knowledge, attitude, and help-seeking behavior.

Stan Kutcher and Yifeng Wei developed a questionnaire to measure mental health knowledge and attitudes toward mental health among adolescents, which was further mentioned as a mental health literacy questionnaire by Carr, Wei, Kutcher, and Heffernan (15). Milin et al. (23) then modified and validated a questionnaire first used by the Youth Opinion Survey on attitudes toward mental health in adolescents. Of all items contained in the questionnaire, this mental health literacy questionnaire could help to address the issue of transitional age adolescents in terms of stress and psychological distress (11). However, the questionnaire has not yet been used widely across Indonesia as part of child and adolescent psychiatric services, mainly due to the absence of an Indonesian language version. This study aimed to translate and assess the validity and reliability of the mental health literacy questionnaires designed to assess mental health knowledge, attitudes, and help-seeking behavior among adolescents in Indonesia.

## Materials and Methods

### Design

This was a cross-sectional study, as part of a project to develop a mental health module for university students involving first-year medical students, and was conducted between October 2020 and January 2021 at the University of Indonesia campuses in Depok and Jakarta, Indonesia. The study was approved by the Cipto Mangunkusumo National Hospital, Jakarta, Indonesia, and ethically approved by the Health Research Ethics Committee, Faculty of Medicine, University of Indonesia, numbered KET-527/UN2.F1/ETIK/PPM.00.02/2020 under protocol number 20-05-0538.

### Participants

Ten experts in child and adolescent psychiatry were included in the study to determine the internal validity of the questionnaires. There is no consensus on how sample size should be measured in psychometric validation studies. Various sources state that it should be 2–20 times the number of items in the questionnaire (24). In this study, to assess construct validity and reliability, sample size was determined by the proportion difference equation *n* = *(Z*α^2^ × *p* × *q)/d*^2^, used primarily for cross-sectional studies (25). In October 2020, all three questionnaires were administered to 68 first-year medical students who were selected randomly from 170 students to meet the minimum sample number required for the study based on the calculation formula. The proportion (p) was assumed to be 20% taken from a previous study on transitional youth mental health problems (26). Meanwhile, q is 1 – p, d is the limit from error, and α is the confidence degree. Therefore, *n* = *(1.96*^2^ × *0.2* × *0.8)/0.1*^2^ = 61.4. We added 10% and rounded the number up to 68 to ensure that the required sample size was met. We conducted the test on first-year medical students, as they are representative of the group of adolescents in the transition to adulthood who require assessment in terms of their mental health literacy and help-seeking behavior.

### Measures

The study used The Mental Health Literacy and Help-Seeking Behavior questionnaires developed by Stan Kutcher, Yifeng Wei, *et al*., consisting of Mental Health Knowledge, Attitude Toward Mental Health, and Help-Seeking Behavior questionnaires (15). All respondents provided their informed consent to complete the questionnaires and several demographic questions. The Mental Health Knowledge questionnaire consisted of 13 items, in which participants were scored one point for correct answers and zero points for wrong or unsure answers. The maximum and minimum total scores were 13 and 0, respectively. The Attitude Toward Mental Health questionnaire consisted of 12 items, with a 5-point Likert Scale ranging from strongly agree to strongly disagree. The maximum and minimum scores were 60 and 12, respectively. The Help-Seeking Behavior questionnaire consisted of 24 items. Results were deemed satisfactory if the participant scored above average, as determined by the pilot mean/median score.

### Translation and Pilot Study

The Mental Health Literacy and Help-Seeking Behavior questionnaires were translated into the Indonesian language by a bilingual psychiatrist. A few adjustments were made in accordance with the input provided by 10 first-year medical student respondents during the trial test of the questionnaires. An additional example was provided for item 7 of the questionnaire, which stated that depression can be treated effectively using alternative treatment, in order to clarify that alternative treatment is not medical treatment, as the questionnaire may be used for students from a non-medical background. The questionnaires were then back translated into English and communicated back to the cross-cultural transition module team, which included the authors of the questionnaires, and then set into the final questionnaire.

### Validity

#### Content Validity

To perform the content validity test, each item of the questionnaire was assessed by 10 experts on child and adolescents psychiatry. The scoring was conducted quantitatively, whereby experts determined its relevance using the following scale: (1) not relevant, (2) quite relevant, (3) relevant, and (4) highly relevant. The scoring sheet was transferred into MS Excel. A score of (1) and (2) were deemed irrelevant and given a value of 0, while (3) and (4) were deemed relevant and given a value of 1. Furthermore, the calculation was based on the Item-Level Content Validity Index (I-CVI) and Scale-Level Content Validity Index (S-CVI). The I-CVI and S-CVI were used to demonstrate validity. S-CVI/Ave, the mean of I-CVI value for each item of the questionnaire, was also used. Polit et al. (27) suggested that with 10 or more experts, there is little need to compute values for k^*^ and that any I-CVI > 0.78 would be considered excellent. Meanwhile, Torkian et al. (28) mention in their study that Content Validity Indexes were considered to be acceptable when I-CVI and S-CVI were at least 0.78 and 0.90, respectively.

#### Construct Validity

The aim of construct validation is to measure whether the instrument is able to capture what it intends to measure (24). This could be by measuring the relation between each item and the total scores of each category to establish its relation to other variables with which it should, theoretically, be associated positively, negatively, or practically not at all. To quantify construct validity, correlation coefficients and confirmatory factor analysis or structural equation modeling, or other statistical evaluations, can be used (29). Construct validity of The Indonesian Mental Health Literacy and Help-Seeking Behavior Questionnaires was measured using the Pearson correlation coefficient values between each item and the total scores of each category and furthermore between each item (30, 31). The Pearson coefficients are classified as medium (0.30–0.49) and large (>0.50) (30). Similar translation adaptation validity studies have used this method (32).

### Reliability

The reliability was determined using the Cronbach's alpha measurement (33). As the questionnaire is a self-report, the inter-rater reliability was deemed unnecessary.

## Results

### Descriptive Findings

The study conducted in October 2020 involved 68 adolescents who were studying as first-year medical students at the University of Indonesia. The mean age was 18.08 for male and 17.81 for female respondents. All respondents were able to understand English and Bahasa Indonesia. Demographic information of the respondents can be found on Table 1.

**Table 1:** Demographic information.

**Age**	**Frequency (*n* = 68)**	**Percentage (%)**
17	12	17.7
18	50	73.5
19	6	8.8
**Gender**		
Male	25	36.8
Female	43	63.2

### Content Validity

The study tested content validity by having 10 experts on child and adolescent psychiatry assess each item of the questionnaire and calculate the I-CVI and S-CVI. The complete I-CVI and S-CVI data are presented in Table 2.

**Table 2:** Validity test of the Indonesian mental health literacy and help-seeking behavior set of questionnaires.

**Mental health knowledge**			**Attitude toward mental health**			**Help-seeking behavior**		
**No**.	**Relevancy**	**I-CVI**	**No**.	**Relevancy**	**I-CVI**	**No**.	**Relevancy**	**I-CVI**
1	10	1.0	1	10	1.0	1	10	1.0
2	7	0.7	2	10	1.0	2	10	1.0
3	10	1.0	3	9	0.9	3	10	1.0
4	9	0.9	4	9	0.9	4	10	1.0
5	10	1.0	5	9	0.9	5	10	1.0
6	8	0.8	6	9	0.9	6	10	1.0
7	8	0.8	7	9	0.9	7	10	1.0
8	8	0.8	8	8	0.8	8	10	1.0
9	9	0.9	9	8	0.8	9	10	1.0
10	7	0.7	10	9	0.9	10	10	1.0
11	8	0.8	11	10	1.0	11	10	1.0
12	10	1.0	12	8	0.8	12	10	1.0
13	9	0.9				13	10	1.0
						14	10	1.0
						15	10	1.0
						16	10	1.0
						17	10	1.0
						18	10	1.0
						19	10	1.0
						20	9	0.9
						21	10	1.0
						22	10	1.0
						23	9	0.9
						24	9	0.9
S-CVI/Ave = mean I-CVI	0.87			0.90			0.99	

*Relevancy, total number of experts scored “relevant” on the item*.

*I-CVI, Item-Level Content Validity Index; S-CVI, Scale-Level Content Validity Index*.

Based on the validity test, the study produced I-CVI scores ranging between 0.7 and 1.0, with S-CVI scores of 0.87, 0.90, and 0.99 on the Mental Health Knowledge, Attitude Toward Mental Health, and Help-Seeking Behavior questionnaires, respectively. The results show that the questionnaires were deemed relevant in measuring the knowledge, attitude, and help-seeking behavior of an individual regarding mental health.

### Construct Validity

For the knowledge questionnaire, overall, each item had a medium correlation (over 0.3) with the exception of item 4. The attitude questionnaire had a medium to large correlation ranging from 0.41 to 0.76, except for item 10. Finally, for the help-seeking behavior questionnaire, most items had a medium to large correlation ranging from 0.30 to 0.69, as seen in Table 3.

**Table 3:** Item-total correlation coefficients for the Indonesian mental health literacy and help-seeking behavior set of questionnaires.

**Questionnaire**	**Item number**	**Item-total correlation**
Knowledge		
	1	0.32^**^
	2	0.32^**^
	3	0.34^**^
	4	0.28^*^
	5	0.51^**^
	6	0.45^**^
	7	0.39^**^
	8	0.38^**^
	9	0.37^**^
	10	0.37^**^
	11	0.53^**^
	12	0.43^**^
	13	0.46^**^
Attitude		
	1	0.41^**^
	2	0.48^**^
	3	0.51^**^
	4	0.63^**^
	5	0.55^**^
	6	0.48^**^
	7	0.59^**^
	8	0.76^**^
	9	0.67^**^
	10	0.25^*^
	11	0.65^**^
	12	0.64^**^
Help-seeking behavior		
	1	0.38^**^
	2	0.52^**^
	3	0.57^**^
	4	0.68^**^
	5	0.63^**^
	6	0.59^**^
	7	0.60^**^
	8	0.68^**^
	9	0.69^**^
	10	0.68^**^
	11	0.68^**^
	12	0.48^**^
	13	0.41^**^
	14	0.32^**^
	15	0.41^**^
	16	0.43^**^
	17	0.39^**^
	18	0.30^*^
	19	0.21
	20	0.33^**^
	21	0.40^**^
	22	0.08
	23	0.22
	24	0.33^**^

** *p-level = 0.01*,

* *p = 0.05*.

### Reliability

A reliability test was performed to identify correlative reliability between items and internal consistency. The study revealed that the Cronbach's alpha values for the reliability test were 0.521, 0.780, and 0.852 for the Mental Health Knowledge, Attitude Toward Mental Health, and Help-Seeking Behavior questionnaires, respectively. The results of the reliability test on each questionnaire are provided in Table 4. The inter-item correlations for each item are presented in Tables 5–7. The mean score for the Mental Health Knowledge questionnaire was 6.97 (SD 2), 47.26 (SD 5.397) for the Attitude Toward Mental Health questionnaire, and 46.66 (SD 6.825) for the Help-Seeking Behavior questionnaire.

**Table 4:** Internal consistency of the Indonesian mental health literacy and help-seeking behavior set of questionnaires.

**Scale**	**Item**	**Corrected-item total correlation**	**Cronbach's alpha if item deleted**	**Cronbach's alpha**
Mental Health Knowledge				0.521
	1	0.103	0.526	
	2	0.182	0.506	
	3	0.232	0.501	
	4	0.033	0.551	
	5	0.303	0.472	
	6	0.251	0.487	
	7	0.159	0.514	
	8	0.274	0.495	
	9	0.129	0.524	
	10	0.193	0.502	
	11	0.334	0.462	
	12	0.340	0.490	
	13	0.291	0.480	
Attitude toward mental health				0.780
	1	0.267	0.781	
	2	0.329	0.775	
	3	0.399	0.767	
	4	0.532	0.754	
	5	0.430	0.764	
	6	0.365	0.770	
	7	0.480	0.758	
	8	0.683	0.736	
	9	0.565	0.749	
	10	0.054	0.811	
	11	0.563	0.751	
	12	0.548	0.752	
Help-seeking behavior				0.852
	1	0.277	0.853	
	2	0.421	0.846	
	3	0.480	0.844	
	4	0.600	0.838	
	5	0.552	0.840	
	6	0.534	0.842	
	7	0.516	0.842	
	8	0.628	0.838	
	9	0.624	0.837	
	10	0.620	0.838	
	11	0.614	0.838	
	12	0.388	0.847	
	13	0.356	0.848	
	14	0.275	0.850	
	15	0.348	0.848	
	16	0.377	0.847	
	17	0.335	0.848	
	18	0.234	0.851	
	19	0.161	0.852	
	20	0.300	0.851	
	21	0.369	0.849	
	22	0.066	0.853	
	23	0.199	0.852	
	24	0.297	0.850	

**Table 5:** Inter-item correlation coefficients for Indonesian adolescents mental health knowledge questionnaire.

**Knowledge item**	**1**	**2**	**3**	**4**	**5**	**6**	**7**	**8**	**9**	**10**	**11**	**12**	**13**
1	1.000	–	–	–	–	–	–	–	–	–	–	–	–
2	0.060	1.000	–	–	–	–	–	–	–	–	–	–	–
3	0.000	−0.143	1.000	–	–	–	–	–	–	–	–	–	–
4	−0.068	−0.018	0.140	1.000	–	–	–	–	–	–	–	–	–
5	0.018	0.241	0.167	−0.071	1.000	–	–	–	–	–	–	–	–
6	0.160	0.173	0.156	−0.176	0.221	1.000	–	–	–	–	–	–	–
7	−0.035	0.182	0.068	−0.168	0.064	0.151	1.000	–	–	–	–	–	–
8	0.144	0.363	0.063	−0.140	0.374	0.139	0.189	1.000	–	–	–	–	–
9	0.068	−0.122	0.015	0.233	0.120	0.033	−0.046	−0.140	1.000	–	–	–	–
10	0.161	0.004	−0.060	0.094	0.052	−0.039	0.065	0.060	0.254	1.000	–	–	–
11	0.090	0.113	0.085	0.011	0.209	0.263	0.307	0.179	−0.051	0.061	1.000	–	–
12	0.041	0.067	0.251	0.084	0.144	0.218	0.022	0.054	0.084	0.094	0.301	1.000	–
13	−0.069	−0.004	0.399	0.306	0.121	0.039	0.099	0.110	0.066	0.085	0.108	0.295	1.000

**Table 6:** Inter-item correlation coefficients for Indonesian adolescents' attitude toward mental health questionnaire.

**Attitude item**	**1**	**2**	**3**	**4**	**5**	**6**	**7**	**8**	**9**	**10**	**11**	**12**
1	1.000	–	–	–	–	–	–	–	–	–	–	–
2	0.363	1.000	–	–	–	–	–	–	–	–	–	–
3	0.257	0.349	1.000	–	–	–	–	–	–	–	–	–
4	0.162	0.100	0.197	1.000	–	–	–	–	–	–	–	–
5	0.152	0.156	0.087	0.35	1.000	–	–	–	–	–	–	–
6	0.120	0.024	−0.126	0.547	0.442	1.000	–	–	–	–	–	–
7	0.295	0.474	0.328	0.198	0.193	−0.072	1.000	–	–	–	–	–
8	0.109	0.138	0.207	0.523	0.482	0.414	0.397	1.000	–	–	–	–
9	0.136	0.267	0.374	0.241	0.259	0.203	0.340	0.581	1.000	–	–	–
10	−0.071	−0.01	0.253	−0.078	0.041	−0.079	0.173	0.017	0.194	1.000	–	–
11	0.146	0.047	0.179	0.617	0.285	0.467	0.301	0.643	0.374	−0.137	1.000	–
12	0.015	0.118	0.225	0.519	0.241	0.416	0.187	0.629	0.409	0.033	0.631	1.000

**Table 7:** Inter-item correlation coefficients for mental health help-seeking behavior of Indonesian adolescents questionnaire.

**Help-seeking item**	**1**	**2**	**3**	**4**	**5**	**6**	**7**	**8**	**9**	**10**	**11**	**12**	**13**	**14**	**15**	**16**	**17**	**18**	**19**	**20**	**21**	**22**	**23**	**24**
1	1.000	–	–	–	–	–	–	–	–	–	–	–	–	–	–	–	–	–	–	–	–	–	–	–
2	−0.082	1.000	–	–	–	–	–	–	–	–	–	–	–	–	–	–	–	–	–	–	–	–	–	–
3	0.151	0.298	1.000	–	–	–	–	–	–	–	–	–	–	–	–	–	–	–	–	–	–	–	–	–
4	0.095	0.591	0.217	1.000	–	–	–	–	–	–	–	–	–	–	–	–	–	–	–	–	–	–	–	–
5	0.082	0.254	0.618	0.443	1.000	–	–	–	–	–	–	–	–	–	–	–	–	–	–	–	–	–	–	–
6	0.237	0.242	0.251	0.542	0.437	1.000	–	–	–	–	–	–	–	–	–	–	–	–	–	–	–	–	–	–
7	0.205	0.265	0.334	0.356	0.418	0.286	1.000	–	–	–	–	–	–	–	–	–	–	–	–	–	–	–	–	–
8	0.205	0.297	0.315	0.406	0.431	0.480	0.345	1.000	–	–	–	–	–	–	–	–	–	–	–	–	–	–	–	–
9	0.234	0.246	0.490	0.382	0.447	0.388	0.590	0.452	1.000	–	–	–	–	–	–	–	–	–	–	–	–	–	–	–
10	0.261	0.247	0.179	0.441	0.292	0.405	0.379	0.654	0.460	1.000	–	–	–	–	–	–	–	–	–	–	–	–	–	–
11	0.257	0.264	0.350	0.385	0.400	0.403	0.342	0.388	0.427	0.501	1.000	–	–	–	–	–	–	–	–	–	–	–	–	–
12	0.334	0.394	0.197	0.338	0.173	0.271	0.233	0.235	0.103	0.0.363	0.421	1.000	–	–	–	–	–	–	–	–	–	–	–	–
13	0.015	0.161	0.217	0.270	0.174	0.348	0.039	0.101	0.219	0.122	0.267	0.051	1.000	–	–	–	–	–	–	–	–	–	–	–
14	0.256	0.147	0.184	0.116	0.019	−0.132	0.422	−0.009	0.188	0.047	0.034	0.080	0.194	1.000	–	–	–	–	–	–	–	–	–	–
15	0.033	0.211	0.122	0.235	0.122	0.072	0.044	0.418	0.179	0.344	0.192	−0.008	0.311	0.287	1.000	–	–	–	–	–	–	–	–	–
16	0.205	0.067	0.200	0.154	0.279	0.149	0.253	0.174	0.380	0.295	0.063	−0.019	0.331	0.485	0.422	1.000	–	–	–	–	–	–	–	–
17	0.181	−0.033	−0.001	0.172	0.070	0.181	0.078	0.389	0.292	0.557	0.341	0.073	0.187	−0.054	0.423	0.338	1.000	–	–	–	–	–	–	–
18	0.026	−0.100	0.042	0.072	0.077	0.061	0.030	0.168	0.115	0.251	0.322	0.006	0.456	0.233	0.215	0.223	0.312	1.000	–	–	–	–	–	–
19	0.131	0.064	−0.053	0.047	−0.053	−0.004	−0.081	0.195	0.013	0.037	0.198	0.147	0.221	0.106	0.241	0.019	0.190	0.285	1.000	–	–	–	–	–
20	0.012	0.220	0.205	0.224	0.205	0.111	0.173	0.172	0.154	0.042	0.133	0.095	0.114	0.144	0.022	0.026	−0.104	0.047	0.113	1.000	–	–	–	–
21	0.089	0.289	0.201	0.294	0.133	0.145	0.182	0.226	0.247	0.115	0.174	0.100	0.150	0.247	0.106	0.076	0.015	0.137	0.201	0.763	1.000	–	–	–
22	−0.151	0.125	0.117	0.127	0.117	0.063	−0.097	0.098	−0.011	−0.106	0.075	−0.056	0.065	−0.045	0.096	−0.076	−0.142	−0.137	−0.051	0.569	0.434	1.000	–	–
23	0.122	0.070	0.166	−0.021	0.166	0.090	0.071	0.139	−0.015	−0.012	0.107	0.156	0.093	0.207	−0.042	0.086	−0.026	0.155	0.419	0.386	0.284	−0.021	1.000	–
24	0.014	0.256	0.088	0.261	0.163	0.129	0.176	0.200	0.129	0.082	0.154	0.139	0.133	0.103	0.068	−0.016	−0.037	0.096	0.073	0.859	0.648	0.489	0.326	1.000

## Discussion

The current study aimed to provide evidence of the validity and reliability of tools for assessing mental health among Indonesian adolescents in three categories: mental health knowledge, attitude toward mental health, and help-seeking behavior. An Indonesian version of the Mental Health Literacy and Help-Seeking Behavior questionnaires, which could be used to assess the mental health literacy and help-seeking behavior of Indonesian adolescents, is yet to exist. Youth well-being is an important component attributing to improved mental health services across the nation. Therefore, by having access to valid and reliable questionnaires, health professionals could better understand the mental health situation among adolescents within the community before conducting further studies or interventions.

### Content Validity

Content validity was assessed based on relevancy, clarity, and ambiguity. For Mental Health Knowledge, the internal validity test showed an I-CVI ranging between 0.7 and 1.0, with a S-CVI of 0.87, meaning that 87% of the items were deemed relevant, clear, and unambiguous by the panel of experts. Similarly, the internal validity test on the questionnaire measuring Attitude Toward Mental Health had an I-CVI of 0.8–1.0, with an S-CVI of 0.90, showing that 90% of the items were also deemed understandable. As for Help-Seeking Behavior questionnaire, each item showed an I-CVI of either 0.9 or 1.0, with an S-CVI of 0.99, meaning that 99% of the items were deemed relevant and clear.

Seven of the 10 experts deemed all items to be appropriate. As the study could be used as the pilot study for further research with similar purposes, there was no precise cutoff point to set the baseline for the questionnaire being considered valid.

### Construct Validity

A construct validity test was conducted to assess whether the questionnaires measured the intended constructs, which were knowledge, attitudes, and help-seeking behavior. To measure whether each questionnaire is homogeneous, we conducted Pearson *r* item-total correlation analysis. The Pearson correlation is used to assess the linear association between variables. Although most items revealed a moderate to high correlation, a few of the items had a low correlation and were statistically insignificant. It has been noted that it would be helpful if further evidence of construct validity could be obtained, for example, convergent evidence and discriminant evidence, which could be retrieved from factor analysis (32, 34).

### Reliability

In order to evaluate the internal consistency of the research tool, the Cronbach's alpha coefficient was used. This method has been the most widely employed method in validation studies (35). An alpha value >0.70 is considered good, whereby when reliability increases, the fraction of a test score attributable to error will decrease. Out of the questionnaires, the Attitude Toward Mental Health questionnaire and Help-Seeking Behavior questionnaire had an alpha value of above 0.70, demonstrating their effectiveness and acceptability.

The set of questionnaires was disseminated to 68 respondents, surpassing the minimum sample number of 62. Wei et al. (36) also suggested in their findings that, in order to examine the internal consistency and the dimensionality of the tool, a sample size of at least 30 individuals may be required. The Cronbach's alpha value for the Mental Health Knowledge questionnaire obtained in this study was 0.521. This value is relatively fair and considered to be low to moderate in terms of reliability, but not unacceptable (37). The English version of the Mental Health Knowledge questionnaire, which was used for a randomized clinical trial on high school students, and which was the reference for the Indonesian questionnaire, demonstrated a Cronbach's alpha of 0.40 (preintervention) and 0.54 (postintervention) (23).

To acquire a higher Cronbach's alpha value, it has been suggested that providing a larger number of items in the scale could be taken into consideration. A low alpha value could be caused by the following reasons: fewer numbers of questions, poor inter-item correlation, or heterogeneous constructs of the items (38). Nevertheless, if the items targeting a unidimensional construct are parallel, one alternative method to enhance the alpha value is to develop a set of highly correlated items in the tool, without unduly increasing the number of items with a lack of inter-item correlation (39).

### Future Appliance and Practical Settings

This validity and reliability study indicates that the translated mental health literacy questionnaires are reasonably valid and can be further applied for wider use in the setting of psychiatric services for adolescents. We also suggest that our study could be used to address the finding made by Vidourek *et al*., in which a lack of support and inadequate treatment from health professionals could increase the risk of students not finishing their studies, which subsequently leads to worsening mental health conditions (40).

Several studies have shown that students, who are in the period of transitioning from adolescents to young adults, tend to seek informal help from relatives or friends, rather than professional help when they encounter psychological distress. This lack of willingness to seek professional support results in higher stress levels and poor academic performance (41, 42). With the existence of these questionnaires in Indonesia, mental health professionals will be able to further understand and gain detailed insights into why individuals are hesitant to seek help.

Currently, according to the Indonesian 2020–2024's Plans of Actions in Mental Health and Psychoactive Drugs, one strategic issue faced by the government is a lack of funding, while not all district authorities have set policies to address mental health issues (43). This study suggests that the questionnaires could be utilized to create a hassle-free experience and are able to penetrate all layers of the community. In the future, these questionnaires could serve as tools for the government and relevant stakeholders to establish affordable mental health policies. Moreover, a study in Indonesia showed that despite the high number of university students suffering from anxiety (95.4%), ~90–96.4% exhibited positive coping strategies, and nearly 50% reported self-harming and had suicidal thoughts (21). We suggest that the tools examined in this study be applied by health facilities in universities to perform initial assessment of first-year students. In the long term, the data obtained can be used as a reference for more effective and accurately targeted interventions to address mental health issues in adolescents.

### Study Limitations

There are several limitations to this study. The sample group for the study was adolescents in the transitional phase who are first-year medical students. In order to further examine the effectiveness of these questionnaires, the coverage could be expanded to include adolescents in different phases and from a general academic background. Furthermore, a test–retest study could also be considered to re-evaluate the set of questionnaires in the future.

## Conclusion

The mental health and wellbeing of adolescents is a dynamic and growing issue that must be addressed properly in the field of psychiatry considering that literacy around the underlying causes, the existence of stigma, and sense of severity could impair an individual's quality of life. The availability of proper means of measurement by the existence of accessible yet valid tools is an integral part of addressing the issue. This is the first set of mental health literacy tools which have been translated and validated into the Indonesian language. From the results of our study, the Indonesian version of the Mental Health Literacy and Help-Seeking Behavior questionnaires is considered to be valid and reliable. These tools are easily understood by all levels of society and could be used to support future studies focusing on mental health literacy among adolescents.

## Data Availability Statement

The raw data supporting the conclusions of this article will be made available by the authors, without undue reservation.

## Ethics Statement

The studies involving human participants were reviewed and approved by Health Research Ethics Committee, Faculty of Medicine, University of Indonesia. Written informed consent to participate in this study was provided by the participants' legal guardian/next of kin.

## Author Contributions

FK, RI, and TW: designed the study and methods. SP, WI, HG, and VP: contributed to the design of the study and review the interpretation of the data. FK and CM: carried out the data collection. FK, CM, GN, and MA: analyzed the data and write the draft of the manuscript. FK, RI, TW, KM, and SP: contributed in finalizing the manuscript. TT, MK and AS: contributed to the interpretation of the data and critically reviewed and approved the final draft of the manuscript. All authors agree to accept responsibility for all aspects of the work.

## Funding

The funding for this study was from the Program Pendanaan Perancangan dan Pengembangan Purwarupa (P5) grant which was granted by the Directorate of Innovation and Science Techno Park Universitas Indonesia.

## Conflict of Interest

The authors declare that the research was conducted in the absence of any commercial or financial relationships that could be construed as a potential conflict of interest.

## Publisher's Note

All claims expressed in this article are solely those of the authors and do not necessarily represent those of their affiliated organizations, or those of the publisher, the editors and the reviewers. Any product that may be evaluated in this article, or claim that may be made by its manufacturer, is not guaranteed or endorsed by the publisher.
