# Supplementary material for: Translation, Validity, and Reliability of Mental Health Literacy and Help-Seeking Behavior Questionnaires in Indonesia
Source: Front Psychiatry. 2022 Jan 18;12:764666. doi: 10.3389/fpsyt.2021.764666 (PMC8804524; doi:10.3389/fpsyt.2021.764666)
Supplement: Supplementary file 1 [file Data_Sheet_1.docx]

Supplementary Material

# Supplementary Tables

**Table 1**. Adolescents’ Mental Health Knowledge Questionnaire

| **No** | **Item** | ***Benar***  **True** | ***Salah***  **False** | ***Tidak Tahu***  **Do not know** |
| --- | --- | --- | --- | --- |
| 1 | *Sedikit rasa cemas dapat membantu Anda meningkatkan kinerja Anda (contoh: dalam kompetisi olah raga, dalam sebuah ujian, dll.).*  A small amount of anxiety is helpful to improving how well you perform (for example: at a sporting event; on a test; etc.). |  |  |  |
| 2 | *Delusi atau waham terjadi ketika seseorang melihat benda yang tidak benar-benar ada atau mendengar hal yang tidak benar-benar ada.*  A delusion occurs when someone sees things that are not really there or hears things that are not really there. |  |  |  |
| 3 | *Tiga gejala umum depresi adalah: gangguan tidur; merasa tidak berharga; memiliki pemikiran ingin mati atau ingin bunuh diri.*  Three common symptoms of Depression are: problems with sleeping; feeling worthless; having thoughts about death or suicide. |  |  |  |
| 4 | *Anoreksia Nervosa DAN Bulimia Nervosa, KEDUANYA merupakan tipe gangguan makan.*  Anorexia Nervosa AND Bulimia Nervosa are BOTH types of eating disorders. |  |  |  |
| 5 | *Orang dengan gangguan jiwa dapat mencapai derajat kesehatan jiwa yang baik.*  People with mental disorders can achieve good mental health. |  |  |  |
| 6 | *Mempunyai pikiran ingin bunuh diri yang secara terus-menerus adalah reaksi normal terhadap situasi stres dalam hidup.*  Having persistent suicidal thoughts is a normal response to a stressful situation in your life. |  |  |  |
| 7 | *Depresi dapat diatasi secara efektif dengan pengobatan alternatif (paranormal, spiritual, herbal, dll).*  Depression can be treated effectively with alternative therapy |  |  |  |
| 8 | *Gangguan Panik, Gangguan Kecemasan Sosial, dan rasa cemas saat ujian semua adalah contoh gangguan jiwa berupa Gangguan Cemas.*  Panic Disorder, Social Anxiety Disorder and Examination Anxiety are all examples of Anxiety Disorders. |  |  |  |
| 9 | *SEMUA hal berikut ini menggambarkan Gangguan Bipolar: Gangguan Bipolar terjadi pada sekitar 1% populasi; terdiri dari episode manik dan episode depresi (KEDUANYA ada); diobati dengan mood stabilizer.*  ALL of the following describe Bipolar Disorder: it affects about 1% of the population; is comprised of BOTH manic and depressive episodes; is treated with mood stabilizers. |  |  |  |
| 10 | *Situasi akademik sehari-hari yang membebani selama perkuliahan dapat menyebabkan post-traumatic stress disorder (PTSD).*  Most stressful events at the university can cause post traumatic stress disorder (PTSD). |  |  |  |
| 11 | *Seseorang dengan Gangguan Pemusatan Pikiran/Hiperaktifitas (GPPH) dapat menjadi sukses secara akademik dengan bantuan obat dan teknik belajar spesifik.*  A person with Attention Deficit Hyperactivity Disorder (ADHD) can often be academically successful with the help of medication and specific learning techniques. |  |  |  |
| 12 | *Latihan mengatur pernapasan adalah teknik yang dapat Anda pelajari untuk membantu Anda menghadapi situasi penuh stress.*  Box breathing is a technique that you can learn to help you deal with stresful situations. |  |  |  |
| 13 | *Tindakan pasangan yang mengisolasi Anda dari keluarga dan teman-teman Anda merupakan bentuk kekerasan dari hubungan.*  Having a partner who isolates you from family or friends may be a warning sign of an abusive relationship. |  |  |  |

**Table 2**. Adolescents’ Attitude Towards Mental Health Questionnaire

| **No** | **Item** | ***Sangat tidak setuju***  **Strongly disagree** | ***Tidak setuju***  **Disagree** | ***Netral***  **Neutral** | ***Setuju***  **Agree** | ***Sangat setuju***  **Strongly agree** |
| --- | --- | --- | --- | --- | --- | --- |
| 1 | *Seseorang yang menjalani pengobatan kesehatan jiwa memiliki kecerdasan yang sama dengan orang lain.*  A person who has received mental health treatment is just as intelligent as the average person. |  |  |  |  |  |
| 2 | *Seseorang yang menjalani pengobatan kesehatan jiwa dapat dipercaya seperti orang lain.*  Someone who has received mental health treatment is just as trustworthy as the average person. |  |  |  |  |  |
| 3 | *Seseorang yang telah sepenuhnya pulih dari gangguan kejiwaan dapat bekerja sebagai guru di sekolah umum.*  It is acceptable that someone who has fully recovered from a mental illness can work as a teacher of young children in a public school. |  |  |  |  |  |
| 4 | *Mendapatkan pengobatan kesehatan jiwa adalah tanda kegagalan atau kelemahan seseorang.*  Receiving mental health treatment is a sign of personal failure or weakness. |  |  |  |  |  |
| 5 | *Penderita gangguan jiwa berat tidak pernah cukup pulih untuk memiliki kualitas hidup yang baik.*  People with severe mental illness can never recover enough to have a good quality of life. |  |  |  |  |  |
| 6 | *Penderita gangguan jiwa patut disalahkan atas kondisi mereka sendiri.*  People with mental illness are to blame for their own condition. |  |  |  |  |  |
| 7 | *Jika rekan saya memberi tahu bahwa ia mengalami gangguan jiwa, saya masih mau bekerja dengannya.*  If my colleague told me they had a mental illness, I would still want to work with them. |  |  |  |  |  |
| 8 | *Jika saya memiliki tetangga dengan gangguan jiwa, saya akan pindah dari lingkungan tersebut.*  If I had neighbors with mental illness, I would move out of that neighborhood. |  |  |  |  |  |
| 9 | *Jika seseorang yang telah sepenuhnya pulih dari gangguan jiwa meminta saya untuk memberikan surat dukungan untuk mendapatkan pekerjaan, saya akan memberikannya.*  If a person who had fully recovered from mental illness asked me for a letter of support to get employment, I would provide a reference. |  |  |  |  |  |
| 10 | *Jika saya mengalami gangguan jiwa, saya tidak akan mengakui keadaan tersebut kepada teman saya karena saya takut akan diperlakukan secara berbeda.*  If I had a mental illness, I would not admit this to any of my friends for fear of being treated differently. |  |  |  |  |  |
| 11 | *Saya akan menerima seseorang yang mendapatkan pengobatan kesehatan jiwa sebagai teman saya.*  I would accept someone who has received mental health treatment as a friend. |  |  |  |  |  |
| 12 | *Saya kurang menghargai seseorang yang mendapatkan pengobatan kesehatan jiwa.*  I would think less of a person who has received mental health treatment. |  |  |  |  |  |

**Table 3**. Mental Health Help-Seeking Behavior of Indonesian Adolescents Questionnaire

| **No** | **Item** | | | | | | |
| --- | --- | --- | --- | --- | --- | --- | --- |
| 1 | *Selama 3 bulan terakhir, apakah Anda pernah berbicara dengan tenaga kesehatan profesional mengenai masalah atau kekhawatiran terkait kesehatan jiwa?*  *□ Saya tidak memiliki masalah atau kekhawatiran mengenai kesehatan jiwa*  *□ Saya sedang menunggu untuk bertemu dengan tenaga kesehatan profesional mengenai masalah atau kekhawatiran terkait kesehatan jiwa*  *□ Saya sudah berbicara dengan seorang tenaga kesehatan profesional mengenai masalah atau kekhawatiran mengenai kesehatan jiwa*  *□ Saya memilih untuk tidak berbicara dengan tenaga kesehatan profesional meskipun saya khawatir dengan kesehatan jiwa saya*  In the last 3 months, have you ever talked to a health professional about any mental health problem or concern?  🞎 I did not have any problem or concern regarding mental health  🞎 I am currently waiting to meet a health professional regarding mental health problem or concern  🞎 I have talked to a health professional regarding mental health problem or concern  🞎 I have decided not to talk to a health professional even though I am concerned about my mental health | | | | | | |
|  | *Pada pertanyaan berikut, kami ingin mengetahui siapakah, jika ada, yang Anda ajak bicara atau Anda mintai bantuan terkait kekhawatiran atau masalah kesehatan jiwa yang Anda alami (dalam 3 bulan terakhir).*  On the next question, we want to know who, if any, have you talked to or asked for help regarding your mental health worries or troubles that you have had (in the past 3 months). Please answer all questions. You can choose more than one option. | | | | | | |
|  |  | ***Meminta bantuan***  **Asked for help** | ***Ingin, tetapi tidak meminta bantuan***  **Wanted to, but did not ask for help** | | | ***Tidak merasa butuh bantuan***  **Did not feel the need to ask** | |
| 2 | *Ibu atau ayah saya (atau yang setara orang tua).*  My mother or father (or anyone equivalent as parents). |  |  | | |  | |
| 3 | *Saudara (adik, kakak, saudara tiri, saudara angkat, dll.).*  Sibling (younger sibling, older sibling, step sibling, foster sibling, etc.) |  |  | | |  | |
| 4 | *Keluarga lain (sepupu, om, tante, kakek, nenek, dll).*  Other relatives (cousin, uncle, aunt, grandfather, grandmother, etc.) |  |  | | |  | |
| 5 | *Teman dekat.*  Close friend. |  |  | | |  | |
| 6 | *Dosen di universitas.*  University lecturer. |  |  | | |  | |
| 7 | *Teman kuliah.*  Coworker. |  |  | | |  | |
| 8 | *Ulama, pendeta, rabi, imam (atau pemimpin spiritual atau agama lainnya).*  Cleric, priest, rabbi, imam (or other spiritual or religious leader). |  |  | | |  | |
| 9 | *Tenaga kesehatan profesional reguler keluarga saya (misalnya, dokter umum atau perawat).*  My family regular health professional (for example, general practitioner or nurse). |  |  | | |  | |
| 10 | *Tenaga kesehatan profesional kejiwaan (seperti konselor, ahli psikologi, psikiater, atau perawat kesehatan jiwa).*  Mental health professional (such as counsellor, psychologist, psychiatrist, or mental health nurse). |  |  | | |  | |
|  | *Pada pertanyaan berikut, kami ingin mengetahui siapakah, jika ada, yang Anda ajak bicara atau Anda mintai bantuan terkait kekhawatiran atau masalah kesehatan jiwa yang Anda alami (di masa yang akan datang).*  Who would you choose as a person to ask for help if you have concern about your mental health in the future? You can choose more than one option. | | | | | | |
|  |  | ***Saya akan meminta bantuan***  **I will ask for help** | | | ***Saya tidak akan meminta bantuan***  **I will not ask for help** | | |
| 11 | *Ibu atau ayah saya (atau yang setara orang tua).*  My mother or father (or anyone equivalent as parents). |  | | |  | | |
| 12 | *Saudara (adik, kakak, saudara tiri, saudara angkat, dll.).*  Sibling (younger sibling, older sibling, step sibling, foster sibling, etc.) |  | | |  | | |
| 13 | *Keluarga lain (sepupu, om, tante, kakek, nenek, dll).*  Other relatives (cousin, uncle, aunt, grandfather, grandmother, etc.) |  | | |  | | |
| 14 | *Teman dekat.*  Close friend. |  | | |  | | |
| 15 | *Dosen di universitas.*  University lecturer. |  | | |  | | |
| 16 | *Teman kuliah.*  Coworker. |  | | |  | | |
| 17 | *Ulama, pendeta, rabi, imam (atau pemimpin spiritual atau agama lainnya).*  Cleric, priest, rabbi, imam (or other spiritual or religious leader). |  | | |  | | |
| 18 | *Tenaga kesehatan profesional reguler keluarga saya (misalnya, dokter umum atau perawat).*  My family regular health professional (for example, general practitioner or nurse). |  | | |  | | |
| 19 | *Tenaga kesehatan profesional kejiwaan (seperti konselor, ahli psikologi, psikiater, atau perawat kesehatan jiwa).*  Mental health professional (such as counsellor, psychologist, psychiatrist, or mental health nurse). |  | | |  | | |
|  | *Secara umum, seberapa setuju atau tidak setuju Anda dengan tiap pernytataan berikut?*  In general, how much do you agree and disagree with each of these statements? | | | | | | |
|  |  | ***Tidak setuju***  **Disagree** | | ***Netral***  **Neutral** | | | ***Setuju***  **Agree** |
| 20 | *Secara umum, meminta bantuan mengenai masalah atau gangguan kesehatan jiwa sangat membantu.*  In general, asking for help for a mental health problem or disorder is helpful. |  | |  | | |  |
| 21 | *Saya merasa nyaman meminta bantuan mengenai masalah atau gangguan kesehatan jiwa.*  I am comfortable asking for help for a mental health problem or disorder. |  | |  | | |  |
| 22 | *Jika saya merasa memiliki masalah kesehatan jiwa atau gangguan jiwa (seperti depresi, gangguan kecemasan sosial, dll), saya akan meminta bantuan.*  If I think I may have a mental health problem or mental disorder (such as depression, social anxiety disorder, etc), I will ask for help. |  | |  | | |  |
| 23 | *Jika saya menduga salah satu teman atau rekan kerja saya membutuhkan bantuan dengan masalah atau gangguan kesehatan jiwa (seperti depresi), saya akan mendorong mereka untuk mencari bantuan.*  If I thought one of my friends or peers needed help with a mental health problem or disorder (such as depression), I would encourage them to seek help. |  | |  | | |  |
| 24 | *Jika saya menduga salah satu anggota keluarga saya membutuhkan bantuan terkait masalah atau gangguan kesehatan jiwa (misalnya depresi), saya akan mendorong mereka untuk mencari bantuan.*  If I suspect one of my family members need help regarding mental health troubles or disorders (such as depression), I will encourage them to search for help. |  | |  | | |  |
